# The Importance of the Study of the Embryonic Metabolome in Assisted Human Reproduction

**DOI:** 10.5935/1518-0557.20140023

**Published:** 2014

**Authors:** Victor C.P.S. Nascimento, Anderson S. Melo, Thalita S. Berteli, Ana K. Bartmann

**Affiliations:** 1 Ana Bartmann Clinic - Ribeirão Preto - SP - Brazil; 2 Ribeirão Preto University - Ribeirão Preto - SP - Brazil; 3 Department of Gynecology and Obstetrics of the clinical section, Ribeirão Preto Medical School - University of São Paulo - Ribeirão Preto - SP - Brazil

**Keywords:** Assisted Reproductive Technologies, epidemiology, IVF, Latin America, multiple birth, outcomes, registry

## Abstract

Since the advent of IVF in 1978, many novel techniques have arisen in Assisted Human Reproduction (AR). Every year thousands of people seek treatment, with success rates ranging from 35% to 40%. The way of assessing an embryo for intrauterine transfer is still carried out by means of morphological parameters, a traditional methodology that brings little information about embryo physiology. Analyses of embryo metabolic activity seem to be excellent predictors of embryo viability and implantation potential. This methodology is still new and experimental, presenting some degree of operational difficulty, for which reason it is not yet performed on a routine basis at major Assisted Reproduction centers. This paper intends to discuss the importance of studying the embryonic metabolome for the selection and transfer of human embryos.

## INTRODUCTION

The use of assisted reproduction techniques has helped thousands of people throughout the world accomplish the project of building a family. Infertile couples use low- and high-complexity protocols to attain their goal of generating a healthy baby at home. However, those methods, whether of high or low complexity, still have success rates lower than 50% at the best reproduction centers (Kissim *et al.*, 2014).

As infertility rates have been growing throughout the world due to factors related to life style, the indiscriminate use of chemicals in diets and medications and the postponement of the desire to procreate, there is an increased demand for assisted reproduction treatments (Yan *et al.*, 2014). Around 5 million children have already been conceived through ART in the world, with an expected increase from 15% to 18% every five years (ESHRE, 2012).

Improvements both in the area of reproductive medicine and obstetrics and neonatology have allowed success rates to increase since the birth of the first test-tube baby in the 70’s. (Rovei *et al.*, 2013) Indeed, some biological parameters still hold true since the first assisted reproduction cycles due to the lack of knowledge about more effective methods. This is the case of the selection of embryos for intrauterine transfer, which until today is done according to merely morphological criteria, with no assessment of embryonic quality (Ferreira *et al.*, 2010).

In order to optimize the embryo morphological evaluation system, several methods have been posited in recent decades. Some methods have proven invasive, such as pre-implantational genetic analysis and lipidomic analysis, while others have not. Among non-invasive methods, most noteworthy are morphokinetic embryo analysis, also known as time-lapse analysis, which uses the continuous monitoring of embryonic development (Wong *et al.*, 2013), the embryo metabolic study, known as metabolome, and the study of embryonic secretions, known as proteomics or secretome (Bromer *et al.*, 2008).

Pre-implantational genetic diagnosis (PGD) uses cytogenetic and/or molecular techniques in order to select embryos that are free of specific genetic conditions (Milachich, 2014; Van Der Aa *et al.*, 2013). Used for the first time in 1990, PGD currently employs several techniques, the most noteworthy being fluorescence in-situ hybridization (FISH) and comparative array genomic hybridization (aCGH) (Handyside *et al.*,1990; Delhanty *et al.*,1993). PGD is performed by removing genetic material from the polar corpuscle, trophectoderm or blastomeres from embryos in the cleavage stage, and it is necessary to use either laser or Tyrone’s acid (Milachich, 2014).

On average, 50% to 60% of embryos on the third day of cultivation show at least one chromosomal change, which is why an embryo biopsy on the 3^rd^ day does not seem to be the best time for obtaining genetic material. On the 5^th^ day of cultivation, when embryos are in the blastocystic stage, genetically modified cells go into apoptosis or become confined to form placental tissue. Accordingly, today, trophectoderm biopsy on the 5^th^ day of cultivation seems to be the best option (Taylor *et al.*, 2014).

Analysis of lipids in embryos, also known as embryonic lipidomic analysis has as its goal to study lipids that are present in the structures of cells by evaluating cellular integrity and functionality (Ferreira *et al.*, 2010). The main lipids found and researched are phospholipids, fatty acids and triacylglycerol (Milne *et al.*, 2006). The study of lipids in human reproduction using matrix-assisted laser desorption/ionization mass spectrometry (MALD-MS) has been helpful in studying the lipidic composition of the embryo’s membrane and its behavior after temperature changes, and its importance is key to studying cell integrity and viability after cryopreservation procedures (Ferreira *et al.*, 2010).

Time-lapse is an embryonic monitoring system where the embryo’s morphokinetic development is evaluated through such parameters as embryonic cleavage time and the kinetics of each stage of its development (Wong *et al.*, 2013), and it can be predicted which embryo under analysis will have the best chance of reaching the blastocystic stage, and the best implantation rate can be obtained (Basile *et al.*, 2013). One of the earliest time-lapse studies was conducted by Payne *et al.* in 1997. That study evaluated the complete sequence of embryonic development stages and the right time of events such as pronuclei formation and embryo cleavage time. Over the last decade, new devices for morphokinetic analysis brought about advancements in the monitoring system, and optimized the embryonic classification and evaluation system (Aparicio *et al.*, 2013). Studying the proteome allows for a description of changes in all proteins expressed and produced from a single genome (Ferreira *et al.*, 2010). In human reproduction, studying the embryonic proteome plays a key role in the non-invasive analysis of embryos (Desai *et al.*, 2006). The enhanced sensitivity of analytical techniques has allowed for the development of new protocols capable not only of drawing the profile of an embryo’s individual proteome, but also of knowing the proteins produced by the embryo in the cultivation drop, the so-called secretome (Soares *et al.*, 2010). Recent studies on protein detection, such as soluble human leukocytic antigen-g (sHLA-G), in the supernatants of cultivation media, has proven to be a non-invasive marker for embryonic selection (Fuzzi *et al.*, 2002). Starting with the activation of the embryonic genome on the 3^rd^ day of cultivation, the embryo is already capable of producing the protein. The sooner a genome is activated and HLA-G protein appears in the cultivation medium, the better will be embryonic quality (Vercammen *et al.*, 2009). Jurisicova *et al.* (1996) were the first to identify sHLA-g, and she realized that its absence is associated with a reduction in embryonic development and a decrease in the gestation rate. It remains undetermined whether pregnancy is induced by an sHLA-G-producing embryo, since the predictive value of the latter in supernatants of the cultivation medium for selecting embryos with good implantation potential remains unknown (Vercammen *et al.*, 2009). The utilization of criteria for embryo metabolic quality to select embryos has been recently suggested (Ferreira *et al.*, 2010; Bromer *et al.*, 2008). The assessment of metabolic quality rather than morphology - or even in association with morphology- may reduce the number of induced cycles and improve the success rate of the assisted reproduction process, i.e., increased pregnancy and live-birth rates (Ferreira *et al.*, 2010; Bromer *et al.*, 2008).

### Metabolome and Assisted Human Reproduction

Metabolome is generally defined as the identification and quantification of all metabolites expressed from a biological sample, such as granulosa cells or embryos, aimed at understanding their functions, their interactions and their contribution to biological processes (Ferreira *et al.*, 2010). In assisted human reproduction techniques, the metabolic profile of embryos has helped the drawing of an embryo development parameter through the expression of proteins, lipids and metabolites in culture media (Ferreira *et al.*, 2010). Non-invasive studies of embryos are, today, a major alternative for embryonic classification and analysis, as the risks linked to exposure and handling are decreased, thus increasing their viability (Seli *et al.*, 2008). However, there are only a few studies addressing this topic, and therefore it cannot yet be said whether an evaluation of such metabolites is directly related to embryonic viability. The difficulty of standardizing analysis techniques, due to the different cultivation protocols employed by clinics, the difficulty of collecting microdrops, the risk of medium contamination and interference of mineral oil, the long time for metabolite extraction and analysis, justify whys and wherefores of their low commercial usage in RA clinics. Several authors (Botros *et al.*, 2008; Ferreira *et al.*, 2010; Gardner *et al.*, 2001) have studied the metabolic profile of developing embryos using non-invasive analytical methods in culture media and observed that there are relationships between consumption/production of such metabolites and embryo viability and pregnancy potential.

The main substances analyzed to assess embryo activity are: pyruvate, lactate and glucose, in addition to amino acids. While lactate is produced, glucose, pyruvate and aminoacids are consumed by the developing embryo. Pyruvate is produced through two pathways to generate ATP in embryonic metabolism: aerobic, through the Krebs cycle, and anaerobic, through the Embden-Meyerhof pathway (Botros *et al.*, 2008). The aerobic metabolism acts during the preimplantation phase (cleavage stage), where pyruvate is the embryo’s main source of energy, whereas glucose uptake is minimal. Turner *et al.* (1994) and Conaghan *et al.* (1993) reported an inverse relationship between pyruvate uptake by the 2-to-8-cell embryo and implantation potential.

The embryo’s capacity to metabolize glucose increases significantly at the transition from morula to blastocyst and seems to reflect the embryo’s developmental potential and its viability (Devreker *et al.*, 2007). Gardner *et al.* (2001) and Renard *et al.* (1980) observed that glucose consumption by D4 was higher in embryos that were able to reach the blastocyst stage. Thus, the use of glycolytic activity as a selection marker resulted in a fourfold increase in pregnancy rates (Lane & Gardner, 1998).

As far as lactate is concerned, considerable amounts are formed during the course of embryo development, especially by means of glycolytic activity (Gott *et al.*, 1990). Based on the fact that one mole of glucose yields two moles of lactate, only 50% of that could be explained in terms of glucose absorption from the culture media; the remaining lactate must be from the embryo’s endogenous sources. Researchers such as Gott *et al.* (1990) suggest the high lactate production by embryos in the preimplantation stage to be an adaptation to culture conditions, and the greater the production the better the adaptive conditions.

In addition to the either isolated or not assessment of glucose, lactate and pyruvate, many papers take into account the assessment of amino acid turnover as a marker of embryo activity and its quality. In a study using the high-performance liquid chromatography technique, Houghton *et al.* (2002) assessed amino acid turnover in a culture media and observed that a decrease in glutamine, arginine, and methionine consumption and a decrease in alanine and asparagine secretion by embryos on the second and third day of culture correlated with an increase in the embryos’ capacity to reach the blastocyst stage. In another study, the same approach was utilized to analyze the changes in the concentration of amino acids secreted by individually cultured human embryos, and an association was reported between decreased glycine and leucine and increased asparagine levels in the culture media of embryos with greater chances of clinical pregnancy and live births (Brison *et al.*, 2004). A recent study using the nuclear magnetic resonance technique showed an association between increased glutamate levels and increased rates of clinical pregnancy and live births (Seli *et al.*, 2008).

### Techniques used to study the embryonic metabolome

Spectrometric and chromatographic techniques are excellent candidates for investigating the metabolic profiles of a biological system, since they are high-yield automated methodologies with rich profiles in terms of information on biological fluids (Botros *et al.*, 2008). The most widely used technique for metabolic analysis is mass spectrometry (MS), which can be coupled with other separate techniques, such as gas chromatography (GC-MS), liquid chromatography (LC-MS) and capillary electrophoresis (CE-MS). Optical spectroscopies such as Fourier transform infrared (FT-IR), Near Infrared spectroscopy (NIR) and Raman provide complementary profiles of the various components within biological fluids, due to the similar physical mechanisms involved in each technique (Botros *et al.*, 2008).

Mass spectrometry (MS) is an analytical technique which can count and measure the mass of a large variety of gas atoms and molecules in ionized forms in a fast, selective, highly sensitive and reliable manner. MS differs from other spectroscopic techniques, such as ultraviolet and infrared rays and nuclear magnetic resonance spectroscopy, which are based on measuring the physical events resulting from the interaction of organic molecules with electromagnetic radiation (Ferreira *et al.*, 2010).

Nuclear magnetic resonance spectroscopy (NMRS) is one of the commonest, most widely used spectroscopic techniques used for analyzing the metabolome, based on the magnetic properties of atomic nuclei. This technique provides a multivariate analysis approach by non-destructively examining tissue and biological fluids (Nicholson *et al.*, 2002). NMRS is a technique that provides a wealth of information, such as the structures of molecules and quantitative analysis, with metabolite identification capabilities with no need for sample preparation (Lindon, 2004; Pauli *et al.*, 2005).

Lastly, that which limits the use of NMRS and MS technologies in clinical applications is their costs, reproducibility and practicality. Although both technologies are excellent candidates for research applications, they are limited due to their size and complexity of operation. An alternative is the optical spectroscopy technique, which is a low-cost technique (Pauli *et al.*, 2005) that measures the interaction of a species with the electromagnetic radiation absorbed, emitted or scattered by the sample analyzed. The term optical spectroscopy encompasses a wide variety of analytical techniques that include ultraviolet, luminescence and dichroism spectroscopy, as well as a subset of techniques referred to as vibrational spectroscopy: Infrared (IR), Near Infrared spectroscopy (NIR) and Raman spectroscopy (Botros *et al.*, 2008).

## CONCLUSION

Metabolic analysis of embryos is a necessary methodology as an adjunct to current embryo selection techniques. Soon, making choices based merely on morphological patterns that say little about actual embryo quality will no longer be accepted. There still remain some problems for the deployment and standardization of techniques to assess embryonic metabolism due to various existing analysis techniques and a large variability of samples (of culture media, lab techniques and stimulation protocols utilized), in addition to the purchasing cost of equipment.

However, the prospects are that after the standardization of a proper technique, it will be internationally accepted and become increasingly simpler, as well as a routine procedure over the course of time.
